# Supplementary material for: The GTPase Nog1 co-ordinates the assembly, maturation and quality control of distant ribosomal functional centers
Source: eLife. 2020 Jan 7;9:e52474. doi: 10.7554/eLife.52474 (PMC6968927; doi:10.7554/eLife.52474)
Supplement: Supplementary file 1. [file elife-52474-supp1.docx]

**Supplementary File 1 - Yeast strains used in this study**

| **Strain name** | **Genotype** | **Origin** |
| --- | --- | --- |
| BY4741 | *MATa ura3 his3 leu2 met15 TRP1* | Euroscarf |
| Nog1 shuffle | *MATa ura3 ade2 leu2 met15 trp1 nog1::HIS3MX6 <pRS316-NOG1>* | This study |
| Nog1 shuffle | *MATa ura3 his3 ade2 leu2 met15 trp1 nog1::KANMX <pRS316-NOG1>* | This study |
| Ssf1-TAP | *MAT*α *his3 leu2 ura3 met15 SSF1-TAP::TRP1* | (Kemmler et al., 2009) |
| Rix1-TAP | *MAT*α *his3 leu2 ura3 met15 RIX1-TAP::TRP1* | (Kemmler et al., 2009) |
| Arx1-TAP | *MAT*α *his3 leu2 ura3 met15 ARX1-TAP::TRP1* | (Kemmler et al., 2009) |
| Kre35-TAP | *MAT*α *his3 leu2 ura3 met15 KRE35-TAP::TRP1* | (Kemmler et al., 2009) |
| Ssf1-TAP Mrt4-GFP | *MAT*α *leu2 ura3 met15 SSF1-TAP::TRP1 MRT4-GFP::HIS3MX6* | (Kemmler et al., 2009) |
| Rix1-TAP Mrt4-GFP | *MAT*α *leu2 ura3 met15 RIX1-TAP::TRP1 MRT4-GFP::HIS3MX6* | (Kemmler et al., 2009) |
| Arx1-TAP Mrt4-GFP | *MAT*α *leu2 ura3 met15 ARX1-TAP::TRP1 MRT4-GFP::HIS3MX6* | (Kemmler et al., 2009) |
| Kre35-TAP Mrt4-GFP | *MAT*α *leu2 ura3 met15 KRE35-TAP::TRP1 MRT4-GFP::HIS3MX6* | (Kemmler et al., 2009) |
| Kre35-TAP *mrt4Δ* | *MAT*α *leu2 ura3 met15 KRE35-TAP::TRP1 mrt4::HIS3MX6* | (Kemmler et al., 2009) |
| Mrt4-GFP | *MATa ura3 leu2 met15 TRP1 MRT4-GFP::HIS3MX6* | Open biosystems |
| Mrt4-GFP *yvh1Δ* | *MATa ura3 leu2 met15 TRP1 MRT4-GFP::HIS3MX6 yvh1::KANMX* | (Kemmler et al., 2009) |
| Rlp24-TAP | *MATa ura3 his3 leu2 ade2 RLP24::TRP1MX6* | (Pertschy et al., 2007) |
| Arx1-GFP | *MATa ura3 leu2 met15 TRP1 ARX1-GFP::HIS3MX* | Open biosystems |
| Nug1-GFP | *MATa ura3 leu2 met15 TRP1 NUG1-GFP::HIS3MX* | Open biosystems |
| Bud20-GFP | *MATa ura3 leu2 met15 TRP1 BUD20-GFP::HIS3MX* | Open biosystems |
| Nog1-GFP | *MATa ura3 leu2 met15 TRP1 NOG1-GFP::HIS3MX* | Open biosystems |
| Tif6-GFP | *MATa ura3 leu2 met15 TRP1 TIF6-GFP::HIS3MX* | Open biosystems |
| Tif6-GFP *yvh1Δ* | *MATa ura3 leu2 met15 TRP1 TIF6-GFP::HIS3MX6 yvh1::KANMX* | (Kemmler et al., 2009) |
| *mrt4Δyvh1Δ* | *MATa ura3 his3 leu2 TRP1 mrt4::KANMX yvh1::KANMX* | (Kemmler et al., 2009) |
| Arx1-GFP *rei1Δ* | *MATa ura3 leu2 met15 TRP1 ARX1-GFP::HIS3MX6 rei1::KANMX* | This study |
| Arx1-GFP Nog1 Shuffle | *MATa ura3 ade2 leu2 met15 trp1 nog1::KANMX <pRS316-NOG1> ARX1-GFP::HIS3MX* | This study |
| Mrt4-GFP Nog1 Shuffle | *MATa ura3 ade2 leu2 met15 trp1 nog1::KANMX <pRS316-NOG1> MRT4-GFP::HIS3MX* | This study |
| Tif6-GFP Nog1 Shuffle | *MATa ura3 ade2 leu2 met15 trp1 nog1::KANMX <pRS316-NOG1> TIF6-GFP::HIS3MX* | This study |
| Kre35-TAP Mrt4-GFP uL10-FLAG | *MAT*α *his3 leu2 ura3 met15 KRE35-TAP::TRP1 MRT4-GFP::HIS3MX uL10-FLAG::KANMX* | This study |
| Kre35-TAP *mrt4Δ* uL10-FLAG | *MAT*α *his3 leu2 ura3 met15 KRE35-TAP::TRP1 mrt4::HIS3MX UL10-FLAG::KANMX* | This study |
