# Supplementary material for: The GTPase Nog1 co-ordinates the assembly, maturation and quality control of distant ribosomal functional centers
Source: eLife. 2020 Jan 7;9:e52474. doi: 10.7554/eLife.52474 (PMC6968927; doi:10.7554/eLife.52474)
Supplement: Supplementary file 2. [file elife-52474-supp2.docx]

**Supplementary File 2 – Plasmids used in this study**

| **Plasmid** | **Relevant markers** | **Source** |
| --- | --- | --- |
| YEP351gal-*NOG1* | *GAL1 NOG1* 2µ L*EU2 AMP* | This study |
| YEP351gal-*NOG1^DN^* | *GAL1 NOG1^G223A^* 2µ L*EU2 AMP* | This study |
| YEP351gal-*NOG1 1-479* | *GAL1 NOG1 1-479* 2µ L*EU2 AMP* | This study |
| YEP351gal-*NOG1^DN^ 1-479* | *GAL1 NOG1^G223A^* 1-479 2µ L*EU2 AMP* | This study |
| YEP351gal-*NOG1-GFP* | *GAL1 NOG1-GFP* 2µ L*EU2 AMP* | This study |
| YEP351gal-*NOG1^DN^-GFP* | *GAL1 NOG1^G223A^-GFP* 2µ L*EU2 AMP* | This study |
| YEP352gal-*NOG1* | *GAL1 NOG1* 2µ *URA3* *AMP* | This study |
| YEP352gal-*NOG1^DN^* | *GAL1 NOG1^G223A^* 2µ *URA3 AMP* | This study |
| YEP352gal-*NOG1^Δ426-537^* | *GAL1 NOG1^Δ426-537^* 2µ *URA3 AMP* | This study |
| YEP351gal-*RPL24-FLAG* | *GAL1 NOG1^G223A^* 2µ L*EU2 AMP* | This study |
| YEP351gal-*RLP24* | *GAL1 RLP24* 2µ L*EU2 AMP* | This study |
| YEP351gal-*RLP24 1-146* | *GAL1 RLP24 1-146* 2µ L*EU2 AMP* | This study |
| YEP351gal-*RLP24 ^Δ91-105^* | *GAL1 RLP24 ^Δ91-105^* 2µ L*EU2 AMP* | This study |
| pYEX4T-1 | *URA3 LEU2* | (Pertschy et al., 2007) |
| pYEX4T-1-*pCUP1-GST-DRG1* | *CUP1-GST-DRG1 URA3 LEU2 AMP* | (Pertschy et al., 2007) |
| pYEX4T-1-*pCUP1-GST-DRG1^DN^* | *CUP1-GST-DRG1^E617Q^ URA3 LEU2 AMP* | (Pertschy et al., 2007) |
| YEP351gal-*DRG1* | *GAL1 DRG1* 2µ L*EU2 AMP* | This study |
| YEP351gal-*DRG1^DN^* | *GAL1 DRG1^E617Q^* 2µ L*EU2 AMP* | This study |
| pRS316-*YVH1* | *YVH1 CEN URA3 AMP* | (Kemmler et al., 2009) |
| pRS315-*MRT4* | *MRT4 CEN LEU2 AMP* | (Kemmler et al., 2009) |
| pRS315-*MRT4^G68E^* | *MRT4 CEN LEU2 AMP* | (Kemmler et al., 2009) |
| pRS315-*MRT4-GFP* | *MRT4-GFP CEN LEU2 AMP* | (Kemmler et al., 2009) |
| pRS315-*MRT4^G68E^-GFP* | *MRT4-GFP CEN LEU2 AMP* | (Kemmler et al., 2009) |
| pRS315-*NMD3^3A^-GFP* | *NMD3^I493A L497A L500A^-GFP CEN AMP* | (Lo et al., 2010) |
| pRS314-*NOG1* | *NOG1 CEN TRP1 AMP* | This study |
| pRS314-*NOG1 1-426* | *NOG1 1-426 CEN TRP1 AMP* | This study |
| pRS314-*NOG1 1-479* | *NOG1 CEN1-479 TRP1 AMP* | This study |
| pRS314-*NOG1 1-579* | *NOG1 1-579 CEN TRP1 AMP* | This study |
| pRS314-*NOG1 1-617* | *NOG1 1-617 CEN TRP1 AMP* | This study |
| pRS315-*NOG1* | *NOG1 CEN LEU2 AMP* | This study |
| pRS315-*NOG1 ^DN^* | *NOG1 ^G223A^ CEN LEU2 AMP* | This study |
| pRS315-*NOG1 1-479* | *NOG1 1-479 CEN LEU2 AMP* | This study |
| pRS315-*NOG1-GFP* | *NOG1-GFP CEN LEU2 AMP* | This study |
