# Supplementary material for: The GTPase Nog1 co-ordinates the assembly, maturation and quality control of distant ribosomal functional centers
Source: eLife. 2020 Jan 7;9:e52474. doi: 10.7554/eLife.52474 (PMC6968927; doi:10.7554/eLife.52474)
Supplement: Supplementary file 3. — The average fraction of >1000 cells showing stronger nuclear fluorescence than cytoplasmic (fn) from three independent experiments. [file elife-52474-supp3.docx]

**Supplementary File 3 – Quantification of microscopy data**

**Average fraction of >1000 cells showing stronger nuclear fluorescence than cytoplasmic (f_n_) from three independent experiments.**

| **Figure** | **Sample** | | **Average f_n_** |  | **Figure** | **Sample** | | **Average f_n_** |
| --- | --- | --- | --- | --- | --- | --- | --- | --- |
| Fig.1D | pGAL-NOG1-GFP | | 0.92 |  | Fig.5B | Arx1-GFP | vector / DMSO | 0.98 |
|  | pGAL-NOG1^DN^-GFP | | 0.07 |  |  | Arx1-GFP | pGAL-NOG1^DN^/DMSO | 0.89 |
| Fig.4B | Rlp24-TAP | pGAL-NOG1 | 0.92 |  |  | Arx1-GFP | vector / DIA | 0.01 |
|  | Rlp24-TAP | pGAL-NOG1^DN^ | 0.95 |  |  | Arx1-GFP | pGAL-NOG1DN/DIA | 0.01 |
| Fig.4C | Bud20-GFP | pGAL-NOG1 | 1.00 |  |  | Tif6-GFP | vector / DMSO | 0.99 |
|  | Bud20-GFP | pGAL-NOG1^DN^ | 1.00 |  |  | Tif6-GFP | pGAL-NOG1^DN^/DMSO | 0.08 |
|  | Nug1-GFP | pGAL-NOG1 | 1.00 |  |  | Tif6-GFP | vector / DIA | 0.12 |
|  | Nug1-GFP | pGAL-NOG1^DN^ | 1.00 |  |  | Tif6-GFP | pGAL-NOG1DN/DIA | 0.02 |
|  | Mrt4-GFP | pGAL-NOG1 | 1.00 |  | Fig. 5D | Arx1-GFP rei1Δ | pGAL-NOG1/REI1 | 0.88 |
|  | Mrt4-GFP | pGAL-NOG1^DN^ | 0.08 |  |  | Arx1-GFP rei1Δ | pGAL-NOG1/REI1-TAP | 0.19 |
|  | Arx1-GFP | pGAL-NOG1 | 1.00 |  |  | Arx1-GFP rei1Δ | pGAL-NOG1^DN^/REI1 | 0.87 |
|  | Arx1-GFP | pGAL-NOG1^DN^ | 0.93 |  |  | Arx1-GFP rei1Δ | pGAL-NOG1^DN^/REI1-TAP | 0.13 |
|  | Nmd3^3A^-GFP | pGAL-NOG1 | 0.88 |  | Fig.6C | Mrt4-GFP | pGAL-NOG1 | 1.00 |
|  | Nmd3^3A^-GFP | pGAL-NOG1^DN^ | 0.04 |  |  | Mrt4-GFP | pGAL-NOG1^DN^ | 0.06 |
|  | Tif6-GFP | pGAL-NOG1 | 0.97 |  |  | Mrt4-GFP | pGAL-NOG1^Δ427-536^ | 0.99 |
|  | Tif6-GFP | pGAL-NOG1^DN^ | 0.06 |  |  | Mrt4-GFP | pGAL-RLP24 | 0.87 |
| Fig.5B | Rlp24-TAP | vector / DMSO | 0.84 |  |  | Mrt4-GFP | pGAL-RLP24^1-146^ | 0.16 |
|  | Rlp24-TAP | pGAL-NOG1^DN^/DMSO | 0.92 |  |  | Mrt4-GFP | pGAL-RLP24^Δ91-105^ | 0.99 |
|  | Rlp24-TAP | vector / DIA | 0.10 |  |  | Arx1-GFP | pGAL-NOG1 | 1.00 |
|  | Rlp24-TAP | pGAL-NOG1^DN^/DIA | 0.06 |  |  | Arx1-GFP | pGAL-NOG1^DN^ | 0.94 |
|  | Bud20-GFP | vector / DMSO | 1.00 |  |  | Arx1-GFP | pGAL-NOG1^Δ427-536^ | 0.99 |
|  | Bud20-GFP | pGAL-NOG1^DN^/DMSO | 1.00 |  |  | Arx1-GFP | pGAL-RLP24 | 0.97 |
|  | Bud20-GFP | vector / DIA | 0.00 |  |  | Arx1-GFP | pGAL-RLP24^1-146^ | 0.08 |
|  | Bud20-GFP | pGAL-NOG1^DN^/DIA | 0.00 |  |  | Arx1-GFP | pGAL-RLP24^Δ91-105^ | 0.99 |
|  | Nug1-GFP | vector / DMSO | 0.99 |  |  | Tif6-GFP | pGAL-NOG1 | 0.97 |
|  | Nug1-GFP | pGAL-NOG1^DN^/DMSO | 1.00 |  |  | Tif6-GFP | pGAL-NOG1^DN^ | 0.06 |
|  | Nug1-GFP | vector / DIA | 0.04 |  |  | Tif6-GFP | pGAL-NOG1^Δ427-536^ | 0.98 |
|  | Nug1-GFP | pGAL-NOG1^DN^/DIA | 0.05 |  |  | Tif6-GFP | pGAL-RLP24 | 0.88 |
|  | Mrt4-GFP | vector / DMSO | 1.00 |  |  | Tif6-GFP | pGAL-RLP24^1-146^ | 0.07 |
|  | Mrt4-GFP | pGAL-NOG1^DN^/DMSO | 0.12 |  |  | Tif6-GFP | pGAL-RLP24^Δ91-105^ | 1.00 |
|  | Mrt4-GFP | vector / DIA | 0.02 |  |  |  |  |  |
|  | Mrt4-GFP | pGAL-NOG1^DN^/DIA | 0.03 |  |  |  |  |  |

| **Figure** | **Sample** | | **Average f_n_** |
| --- | --- | --- | --- |
| Fig.6D | Mrt4-GFP nog1Δ | NOG1/DMSO | 0.96 |
|  | Mrt4-GFP nog1Δ | NOG1^Δ1-479^/DMSO | 0.96 |
|  | Mrt4-GFP nog1Δ | NOG1/DIA | 0.15 |
|  | Mrt4-GFP nog1Δ | NOG1Δ1-479/DIA | 0.09 |
|  | Arx1-GFP nog1Δ | NOG1/DMSO | 0.84 |
|  | Arx1-GFP nog1Δ | NOG1^Δ1-479^/DMSO | 0.62 |
|  | Arx1-GFP nog1Δ | NOG1/DIA | 0.30 |
|  | Arx1-GFP nog1Δ | NOG1Δ1-479/DIA | 0.32 |
|  | Tif6-GFP nog1Δ | NOG1/DMSO | 0.90 |
|  | Tif6-GFP nog1Δ | NOG1^1-479^/DMSO | 0.98 |
|  | Tif6-GFP nog1Δ | NOG1/DIA | 0.09 |
|  | Tif6-GFP nog1Δ | NOG1^1-479^/DIA | 0.23 |
| Fig.7A | Mrt4-GFP | YVH1 | 1.00 |
|  | Mrt4-GFP | vector | 0.05 |
|  | Mrt4-GFP | pGAL-NOG1^DN^ | 0.08 |
|  | Mrt4^G68E^-GFP | YVH1 | 0.99 |
|  | Mrt4^G68E^-GFP | vector | 1.00 |
|  | Mrt4^G68E^-GFP | pGAL-NOG1^DN^ | 1.00 |
|  | Tif6-GFP | MRT4/YVH1 | 1.00 |
|  | Tif6-GFP | MRT4/vector | 0.10 |
|  | Tif6-GFP | MRT4/pGAL-NOG1^DN^ | 0.07 |
|  | Tif6-GFP | MRT4^G68E^/YVH1 | 0.98 |
|  | Tif6-GFP | MRT4^G68E^/vector | 0.90 |
|  | Tif6-GFP | MRT4^G68E^/pGAL-NOG1^DN^ | 0.09 |
